# Cardiac arrest in infants, children, and adolescents: long-term emotional and behavioral functioning

**DOI:** 10.1007/s00431-016-2728-4

**Published:** 2016-05-14

**Authors:** Lennart van Zellem, Elisabeth M. Utens, Marlous Madderom, Jeroen S. Legerstee, Femke Aarsen, Dick Tibboel, Corinne Buysse

**Affiliations:** Intensive Care and Department of Pediatric Surgery, Erasmus MC-Sophia Children’s Hospital, Wytemaweg 80, 3015 CN Rotterdam, The Netherlands; Department of Child and Adolescent Psychiatry/Psychology, Erasmus MC-Sophia Children’s Hospital, Rotterdam, The Netherlands

**Keywords:** Heart arrest, Follow-up studies, Psychopathology, Pediatrics

## Abstract

Very little is known about the psychological consequences of a cardiac arrest (CA) during childhood. Our aim was to assess long-term emotional and behavioral functioning, and its predictors, in survivors of CA in childhood. This long-term follow-up study involved all consecutive infants, children, and adolescents surviving CA in a tertiary-care university children’s hospital between January 2002 and December 2011. Emotional and behavioral functioning was assessed with the Child Behavior Checklist (CBCL), Teacher’s Report Form (TRF), and Youth Self-Report (YSR). Of the eligible 107 CA survivors, 52 patients, parents, and/or teachers filled out online questionnaires. Compared with normative data, parents and teachers reported significantly more attention and somatic problems (age range 6–18 years). Parents also reported more attention problems for age range 1.5–5 years. Twenty-eight percent of the children (*n* = 14) scored in the psychopathological range (i.e., for age range 1.5–18 years; *p* < 0.001) according to parent reports. Male gender, older age, and basic life support were significantly related to worse scores on the scales internalizing problems, externalizing problems, and total problems and subscale attention problems.

*Conclusion*: Long-term deficits in attention and somatic complaints were reported. Attention problems after childhood CA can interfere with school performance. Long-term follow-up with neuropsychological assessment should be organized.

**Table:** 

**What is known:** • *Critical illness has a significant influence on the presence of long-term emotional and behavioral problems.* • *Long-term emotional and behavioral problems have been described for various groups of critically ill children such as congenital heart disease, meningococcal septic shock, and neonatal asphyxia.*
**What is new:** • *This is the first study that addresses long-term emotional and behavioral problems in a relatively large consecutive series of children and adolescents surviving cardiac arrest.* • *Long-term deficits in attention and somatic complaints were reported.*

**Electronic supplementary material:**

The online version of this article (doi:10.1007/s00431-016-2728-4) contains supplementary material, which is available to authorized users.

## Introduction

Survival rate after cardiac arrest (CA) in children is low and dependent on the location of the arrest [9, 15, 23]. Due to hypoxic-ischemic brain injury, CA survivors show long-term physical and neuropsychological impairments [14, 16]. Additionally, these survivors may suffer from the impact of neuropsychological sequelae of the hypoxic-ischemic event on emotional and behavioral functioning. Moreover, the CA itself and the hospital-related experiences (including their pre-existing disease) may have an overwhelming emotional impact, as children and parents may realize that they/their child would have died if no resuscitation was applied.

Very little is known about the long-term psychological consequences of CA during childhood, especially regarding emotions and behavior. This is a neglected field of research. Emotional and behavioral problems can be classified into internalizing problems (intrapsychic problems, such as anxiety, depression, withdrawn behavior, and somatic complaints), externalizing problems (conflicts with rules or others), and social, attention, and thought problems [2]. Long-term emotional and behavioral problems have been described for various groups of critically ill children. For example, in children with congenital heart disease (ConHD), attention problems, anxiety, and depression, among other problems have been reported [29]. In survivors of meningococcal septic shock, long-term assessment showed more parent-reported somatic complaints [27]. After neonatal asphyxia, long-term elevated levels of hyperactivity and the presence of autism have been found [24].

Until now, only one small study by Morris et al. reported on emotional and behavioral outcome after CA, as part of a neuropsychological outcome study [16]. These researchers studied 25 children at least 1 year after CA and reported that few children (number unknown) scored at or lower than 1 SD below the normative population mean of the Child Behavior Checklist (CBCL), a criterion they used as a deficit score. Children showed internalizing problem behavior, and more physically impaired children had worse scores on the hyperactivity scale. As detailed description is lacking and 80 % of these children had a ConHD, it is unknown whether these results can be generalized to all CA survivors.

To bridge this gap in knowledge, the aim of the present study was to systematically investigate long-term emotional and behavioral outcomes, and their predictors, in a consecutive series of survivors of CA in childhood. A multi-informant approach (parents’, teachers’, self-report) was used, comprising internationally validated and well-known psychological questionnaires. We hypothesized that survivors of CA in childhood have long-term emotional and behavioral problems.

## Material and methods

This study was performed at the intensive care unit (ICU) of the Erasmus MC-Sophia Children’s Hospital in Rotterdam. This is the only university specialized pediatric ICU in this region with approximately 4.2 million inhabitants, representative of the Dutch population.

The Erasmus MC Ethical Review Board approved the study protocol (NL 39084.078.12) in accordance with Dutch laws and regulations and international conventions, such as the Declaration of Helsinki.

### Patient sample

The target population consisted of all consecutive surviving patients aged 0–18 years with a CA between January 2002 and December 2011, who were admitted to the ICU of the Erasmus MC-Sophia Children’s Hospital.

CA was defined as absent pulse rate or the need for cardiac compressions. Cardiopulmonary resuscitation (CPR) was defined as “basic life support” (BLS), in line with the European Resuscitation Council Guidelines for pediatric life support, and if needed, followed by “advanced pediatric life support” (APLS) [5].

All CA data were retrospectively collected. Data were derived from ambulance registration forms, clinical and electronic medical records, and CA registration forms. We collected (1) basic patient characteristics, (2) CA characteristics (e.g., location, rhythm, etiology), and (3) outcome (mortality). Additionally, medical records were retrospectively analyzed if the health status prior to the CA was related to the cause of the arrest (i.e., cardiac, respiratory, or other).

Eligible for this study were (1) children resuscitated in-hospital (e.g., emergency department, ward, ICU); (2) children resuscitated in a regional hospital or other university hospital, and after return of spontaneous circulation (ROSC) subsequently admitted to the ICU of our hospital; and (3) children resuscitated out-of-hospital and subsequently admitted to our ICU. Neonates resuscitated at the hospital’s neonatal intensive care unit (NICU) or in another hospital and subsequently admitted to the NICU of our hospital were excluded.

#### Procedure

In May 2013, children and parents (including one caregiver) were invited to participate 2 to 11 years after ICU discharge. Informed consent was obtained from all individual participants (their parents, and children (if ≥12 years)) included in the study. Families were invited to complete online questionnaires on emotional and behavioral functioning. Choice of respondent (mother, father, or caregiver) was left to the parents themselves. Parents and children were explicitly instructed to complete questionnaires separately. Parents were asked to deliver an invitation to their child’s teacher to fill out an online questionnaire.

Emotional and behavioral problems were assessed with Dutch versions of the internationally well-known CBCL, Teacher’s Report Form (TRF), and Youth Self-Report (YSR) to obtain parent, teacher, and self-reports, using parallel standardized questionnaires with overlapping items [2]. These questionnaires are recommended by the American Heart Association [12]. For preschool children (1.5–5 years), the CBCL 1.5–5 and Caregiver-TRF (C-TRF) were used. For school-aged children (6–18 years), the CBCL 6–18 and TRF 6–18 were used. The YSR was completed by adolescents aged 11–18 years.

Items of these questionnaires were rated on a three-point scale (0 = not true; 1 = somewhat or sometimes true; 2 = very true/often true). The preschool forms consist of six subscales and the school age forms of eight subscales. These subscales can be combined in an internalizing problem scale, externalizing problem scale, and an overall total problem scale. Higher scores indicate higher problem levels. Complete datasets with corresponding age ranges of representative normative data were available and consisted of the following large representative samples of the general Dutch population: (1) CBCL 1.5–5 years, 532 children; (2) CBCL 6–18 years, 1451 children; (3) C-TRF, 346 children; (4) TRF 6–18 years, 1016 children; and (5) YSR 11–18 years, 731 children [22].

All psychological questionnaires have adequate psychometric properties [2].

### Predictors

The following predictors were tested: gender, age at ICU admission, age at follow-up, type of CPR (BLS/APLS), location (in-hospital/out-of-hospital), CA-related pre-existing medical condition, present health status, and socioeconomic status (SES) at follow-up.

Present health status was defined as suffering from comorbidity at follow-up, not related to the CA. SES at time of follow-up was based on parents’ occupation and categorized as “low” (elementary occupations), “middle” (“middle” occupations), or “high” (“highest” scientific occupations) conforming to the Dutch standard classification [7]. The highest occupation of both parents was used.

### Statistical analyses

Patient and CA characteristics of participants and nonparticipants were examined with independent sample *t* tests for normally distributed continuous data and Mann-Whitney *U* test for nonnormally distributed data. Fisher’s exact test was used for comparison of dichotomous data.

Normality of data was examined with the Kolmogorov-Smirnov test. To compare CA survivors with normative data, the Welch’s *t* test (for unequal variances) was performed for normally distributed data or the Mann-Whitney *U* test for nonnormally distributed data. Cohen’s *d* effect sizes (ES) were also calculated (an effect size of ≤0.49 is considered small, 0.50–0.79 medium, and ≥0.80 large) [8]. The Kruskal-Wallis test served to test the differences in responses between caregivers (mother, father, and caregiver).

To distinguish problem children (i.e., scoring in the psychopathological range) from nonproblem children, 90th percentiles of the cumulative frequency distributions of total problem scores obtained from the reference groups (gender and age specific) were chosen as the cutoffs [2]. Proportions of children scoring in the psychopathological range in the CA group were compared with the normative group using the binomial test. Predictors of outcome were examined with Spearman correlations for continuous variables, Mann-Whitney *U* test for dichotomous variables, and the Kruskal-Wallis test for ordinal variables. The influence of putative predictor variables was only tested for internalizing, externalizing, and total problem scores and subscales for which mean scores were significantly different from the normative population.

All analyses were performed with SPSS 21.0 for Windows (SPSS, Inc., Chicago, IL). Statistical significance was considered with two-tailed *p* values of <0.05.

## Results

**Fig. 1:**
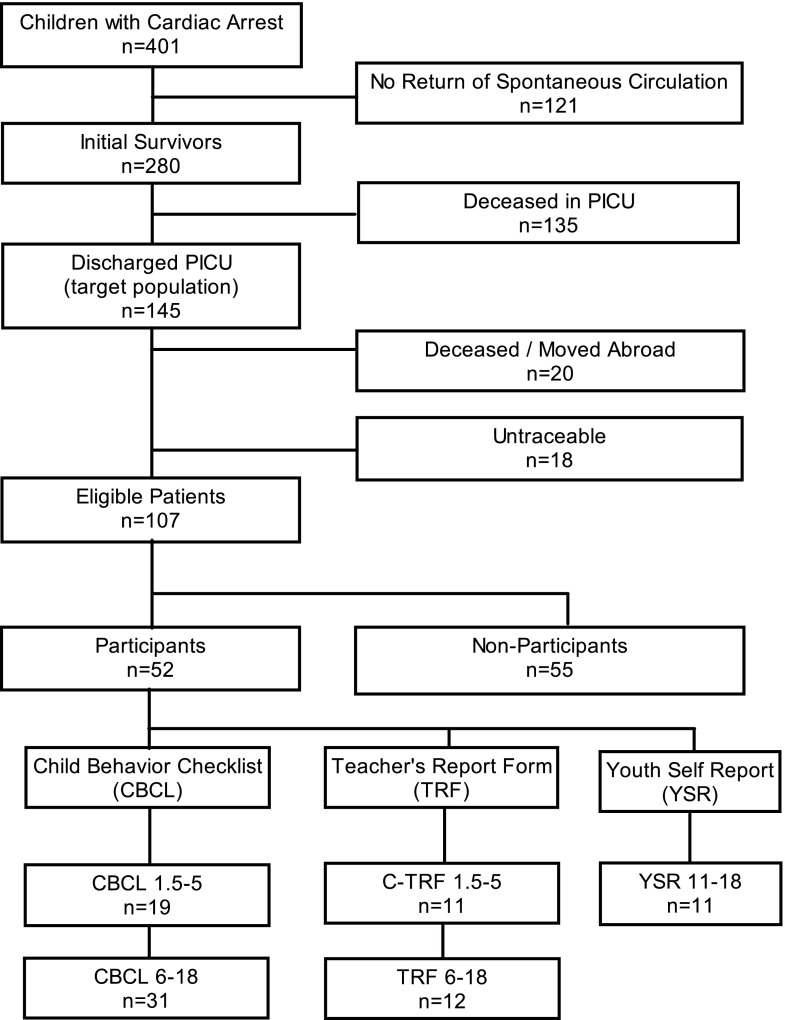
Flowchart of patient inclusion. Note: Two parents did not complete the CBCL, but their child completed the YSR

Our target population consisted of 145 surviving patients, 38 (26 %) of whom were deceased or lost to follow-up (13 died after hospital discharge, 7 moved abroad, 18 untraceable) (Fig. 1). Causes of death after hospital discharge were another CA without ROSC (*n* = 3), underlying disease (*n* = 2), severe cerebral damage (*n* = 1), or unknown (*n* = 7).

Of 107 eligible patients, 52 (49 %) participated. Nonparticipants refused participation due to practical reasons (*n* = 20) or emotional reasons (*n* = 14, e.g., too anxious/confronting). Reasons for nonparticipation were unknown for 21 eligible patients. Patient characteristics of participants versus nonparticipants differed significantly on SES (Table 1).

**Table 1:** Characteristics of participants and nonparticipants

	Participants (*n* = 52 )		Nonparticipants (*n* = 55)		*p* value
	*n* ^a^		*n* ^a^		
Age at ICU admission (months)	52	6.3 (0–193.3)	55	6.5 (0–204.2)	0.267
Male gender	52	28 (54 %)	55	36 (65 %)	0.242
Advanced pediatric life support (APLS)	52	30 (58 %)	55	38 (69 %)	0.235
Out-of-hospital arrest	52	22 (42 %)	55	17 (31 %)	0.235
Bystander CPR	52	51 (98 %)	52	51 (98 %)	1.00
Initial rhythm nonshockable	41	35 (85 %)	51	44 (86 %)	1.00
Etiology					
Cardiac	52	20 (38 %)	55	18 (33 %)	0.552
Arrhythmia	20	6 (30 %)	18	5 (28 %)	
Cardiomyopathy	20	2 (10 %)	18	3 (17 %)	
Hypovolemic shock	20	2 (10 %)	18	1 (6 %)	
Obstructive shock	20	2 (10 %)	18	1 (6 %)	
Septic shock	20	4 (20 %)	18	3 (17 %)	
Other	20	4 (20 %)	18	5 (28 %)	
Respiratory	52	24 (46 %)	55	34 (62 %)	0.123
Aspiration	24	0 (0 %)	34	2 (6 %)	
Bronchomalacia/bronchospasm	24	4 (17 %)	34	3 (9 %)	
Bleeding	24	0 (0 %)	34	2 (6 %)	
Congenital	24	2 (8 %)	34	2 (6 %)	
Drowning	24	9 (38 %)	34	6 (18 %)	
Insufficiency/infection	24	2 (8 %)	34	7 (21 %)	
Intubation related	24	5 (21 %)	34	7 (21 %)	
Obstruction other	24	1 (4 %)	34	1 (3 %)	
Pulmonary hypertension	24	0 (0 %)	34	1 (3 %)	
Other	24	1 (4 %)	34	3 (9 %)	
Neurologic		0 (0 %)		1 (2 %)	1.00
ALTE/SIDS		5 (10 %)		1 (2 %)	0.106
Other/unknown		3 (6 %)		1 (2 %)	0.354
Pre-existing medical condition^b^	52	28 (54 %)	55	27 (49 %)	0.700
Cardiac	28	16 (57 %)	27	14 (52 %)	0.789
Respiratory	28	9 (32 %)	27	11 (41 %)	0.582
Neurologic	28	0 (0 %)	27	1 (4 %)	0.491
Other	28	3 (11 %)	27	1 (4 %)	0.611
Present health status					
Actual comorbidity	52	30 (58 %)	–	–	–
Mild therapeutic hypothermia^c^	52	8 (15 %)	55	12 (22 %)	0.462
Socioeconomic status at follow-up					
Level 1: “low”	52	4 (8 %)	55	14 (25 %)	**0.019**
Level 2: “middle”	52	23 (44 %)	55	29 (53 %)	0.441
Level 3: “high”	52	25 (48 %)	55	12 (22 %)	**0.005**
Age at follow-up (months)	52	103.3 (28.5–220.7)	–	–	–

All data are presented as “number of subject (%)”, except age which is presented as “median (range).” Socioeconomic status (SES) of nonparticipants at time of follow-up was calculated based on a combined status score of the Netherlands Institute for Social Research based on home address [7]. This score consisted of average income in the neighborhood, percentage of people with low income, percentage of less educated people, and percentage of people not working. A status score of 0 (±1.16 SD) was classified middle SES, <−1.16 was classified low SES, and >+1.16 was classified high SES

*CPR* cardiopulmonary resuscitation, *ICU* intensive care unit, *n* number

^a^Number of subjects for which the variable was obtained

^b^Children with a pre-existing medical history which was the cause of the CA

^c^Children treated with mild therapeutic hypothermia

### Emotional and behavioral functioning

The Kolmogorov-Smirnov test indicated that the data were nonnormally distributed; therefore, the Mann-Whitney *U* test was used for comparisons with normative data.

Compared with normative data, parents’ reports (CBCL) showed significantly more attention problems (1.5–5 and 6–18 years; medium ES) and more somatic complaints (6–18 years; large ES) (Tables 2 and 3). Teachers also reported significantly more somatic complaints and attention problems (inattention) in children aged 6–18 years (medium/large ES; Table 3). On item level, the somatic problems most frequently reported were headache and abdominal pain. On self-reports, children reported significantly less social problems than the healthy peers (medium ES).

**Table 2:** Parent and teacher-reported emotional and behavioral functioning: age range 1.5*–*5 years

	Patients Mean (SD)	Norm Mean (SD)		
CBCL (1.5–5 years)	*n* = 19	*N* = 532	*p* value	Cohen’s *d*
Internalizing	9.68 (8.0)	7.86 (5.9)	0.560	0.30
Emotionally reactive	3.05 (3.2)	2.80 (2.5)	0.811	0.10
Anxious/depressed	1.84 (2.1)	1.81 (1.8)	0.859	0.02
Somatic complaints	2.58 (2.9)	1.98 (2.0)	0.604	0.30
Withdrawn	2.21 (2.7)	1.27 (1.4)	0.116	0.64
Sleep problems	2.32 (2.5)	2.18 (2.1)	0.946	0.07
Externalizing	15.37 (12.0)	12.51 (7.2)	0.566	0.38
Attention problems	3.68 (2.9)	2.29 (1.8)	**0.038**	0.74
Aggressive behavior	11.68 (9.3)	10.22 (6.1)	0.835	0.23
Posttraumatic stress problems	2.58 (2.3)	1.99 (1.6)	0.356	0.36
Total problem score	36.37 (25.9)	30.58 (17.1)	0.504	0.33
Deviant range (%)^a^	42 %	10 %	**<0.001**	
C-TRF (1.5–5 years)	*n* = 11	*N* = 346	*p* value	Cohen’s *d*
Internalizing	4.55 (3.3)	5.54 (5.8)	0.954	0.17
Emotionally reactive	0.82 (1.5)	1.51 (2.0)	0.153	0.35
Anxious/depressed	1.18 (1.1)	1.60 (2.0)	0.872	0.21
Somatic complaints	0.45 (0.8)	0.41 (0.9)	0.778	0.04
Withdrawn	2.09 (2.1)	2.03 (2.4)	0.667	0.02
Externalizing	9.09 (10.7)	7.69 (9.5)	0.610	0.15
Attention problems	4.18 (5.1)	2.87 (3.5)	0.478	0.36
Aggressive behavior	4.91 (6.5)	4.82 (6.7)	0.768	0.01
Posttraumatic stress problems	1.55 (1.1)	1.26 (1.5)	0.222	0.19
Total problem score	18.55 (16.5)	17.36 (17.5)	0.662	0.07
Deviant range (%)^a^	9 %	10 %	0.697	

Internalizing problem scale: reflecting intrapsychic problems. Externalizing problem scale: reflecting conflicts with other people or rules. Cohen’s *d*’s are presented as absolute numbers. According to Cohen’s criteria, an effect size of ≤0.49 is considered small, 0.50–0.79 medium, and ≥0.80 large [8]. Higher scores implicate more psychological problems. Mean scale scores were not significantly different between respondents on the CBCL 1.5–5 (mothers *n* = 16, fathers *n* = 3, *p* > 0.05)

*CBCL* Child Behavior Checklist, *C-TRF* Caregiver-TRF

^a^Age and gender specific 90th percentile cutoff scores of the reference group were used

**Table 3:** Parent, teacher, and self-reported emotional and behavioral functioning: age range 6–18 years

	Patients Mean (SD)	Norm Mean (SD)		
CBCL (6–18 years)	*n* = 31	*N* = 1451	*p* value	Cohen’s *d*
Internalizing	8.16 (8.8)	6.64 (5.7)	0.918	0.26
Anxious/depressed	2.55 (3.6)	3.02 (3.0)	0.083	0.16
Withdrawn/depressed	2.23 (2.6)	2.11 (2.2)	0.801	0.05
Somatic complaints	3.39 (4.1)	1.51 (2.0)	**0.003**	0.90
Social problems	2.84 (3.6)	2.06 (2.3)	0.530	0.34
Thought problems	2.23 (3.1)	1.83 (2.1)	0.988	0.19
Attention problems	6.03 (5.0)	3.77 (3.2)	**0.015**	0.70
Externalizing	6.00 (6.6)	6.32 (5.9)	0.446	0.05
Rule-breaking behavior	1.29 (1.6)	2.01 (2.3)	0.092	0.31
Aggressive behavior	4.71 (5.6)	4.32 (4.2)	0.789	0.09
Posttraumatic stress problems	4.77 (4.6)	3.90 (3.3)	0.473	0.26
Total problem score	28.87 (25.5)	23.91 (16.7)	0.651	0.29
Deviant range (%)^a^	19 %	10 %	0.083	
TRF (6–18 years)	*n* = 12	*N* = 1016	*p* value	Cohen’s *d*
Internalizing	5.83 (8.5)	5.21 (5.7)	0.870	0.11
Anxious/depressed	2.33 (4.1)	2.77 (3.3)	0.194	0.13
Withdrawn/depressed	2.08 (3.2)	1.98 (2.5)	0.684	0.04
Somatic complaints	1.42 (2.3)	0.46 (1.2)	**0.010**	0.78
Social problems	1.17 (1.2)	1.58 (2.4)	0.860	0.17
Thought problems	0.33 (0.7)	0.45 (1.1)	0.958	0.11
Attention problems	13.00 (8.8)	8.21 (8.6)	**0.036**	0.56
Inattention	9.17 (6.0)	4.86 (5.2)	**0.007**	0.83
Hyperactivity-impulsivity	3.83 (4.2)	3.35 (4.4)	0.466	0.11
Externalizing	4.08 (5.8)	4.22 (6.7)	0.683	0.02
Rule-breaking behavior	1.42 (2.9)	1.27 (2.2)	0.900	0.07
Aggressive behavior	2.67 (3.7)	2.94 (4.9)	0.635	0.05
Posttraumatic stress problems	3.00 (3.6)	2.92 (3.0)	0.904	0.03
Total problem score	24.75 (16.1)	20.36 (19.5)	0.195	0.23
Deviant range (%)^a^	0 %	10 %	0.282	
YSR (11–18 years)	*n* = 11	*N* = 731	*p* value	Cohen’s *d*
Internalizing	11.73 (8.4)	10.25 (7.0)	0.640	0.21
Anxious/depressed	3.82 (4.3)	4.22 (3.7)	0.423	0.11
Withdrawn/depressed	3.18 (2.7)	3.00 (2.3)	0.997	0.08
Somatic complaints	4.73 (3.7)	3.03 (2.6)	0.083	0.64
Social problems	1.82 (2.4)	3.27 (2.4)	**0.027**	0.60
Thought problems	2.09 (1.7)	3.12 (2.7)	0.279	0.38
Attention problems	4.36 (2.7)	5.12 (3.7)	0.471	0.25
Externalizing	7.64 (4.3)	9.21 (6.4)	0.518	0.25
Rule-breaking behavior	2.82 (1.2)	3.88 (3.1)	0.389	0.35
Aggressive behavior	4.82 (3.7)	5.34 (3.9)	0.659	0.13
Posttraumatic stress problems	5.91 (4.3)	5.99 (3.7)	0.835	0.02
Total problem score	31.00 (14.4)	35.06 (18.2)	0.461	0.22
Deviant range (%)^a^	0 %	10 %	0.314	

Internalizing problem scale: reflecting intrapsychic problems. Externalizing problem scale: reflecting conflicts with other people or rules. Cohen’s *d*’s are presented as absolute numbers. According to Cohen’s criteria, an effect size of ≤0.49 is considered small, 0.50–0.79 medium, and ≥0.80 large [14]. Higher scores implicate more psychological problems. Mean scale scores were not significantly different between respondents on the CBCL 6–18 (mothers *n* = 22, fathers *n* = 8, *p* > 0.05 (including caregiver *n* = 1, *p* > 0.05))

*CBCL* Child Behavior Checklist, *TRF* Teacher’s Report Form, *YSR* Youth Self-Report

^a^Age and gender specific 90th percentile cutoff scores of the reference group were used

According to parent reports, the percentages of patients scoring in the psychopathological range were 28 % (*n* = 14; *p* < 0.001) when combining both age groups (i.e., for age range 1.5–18 years). Subanalyses of the different age ranges showed that the percentages in the deviant range were specifically elevated for children aged 1.5–5 (42 %, *p* < 0.001), whereas for the older age range (6–18 years), a nonsignificant trend (*p* = 0.083) was found. When combining both age groups of the teacher reports, no significant differences were found (psychopathological range 4 % (*n* = 1); *p* = 0.315) (Tables 2 and 3).

Mean scale scores were not significantly different between mothers versus fathers on the CBCL 1.5–5 (mothers *n* = 16, fathers *n* = 3) and the CBCL 6–18 (mothers *n* = 22, fathers *n* = 8, and caregiver *n* = 1).

### Predictors of outcome

Univariate analysis showed that, on parent reports for 1.5–5-year-old children, older age at ICU admission was a significant predictor of more externalizing problems (*ρ* = 0.505, *p* = 0.027) and total problem scores (*ρ* = 0.509, *p* = 0.026) (Supplemental Table 1). This was also found on the subscale attention problems (*ρ* = 0.523, *p* = 0.022).

BLS/APLS was a significant predictor of internalizing (*Z* = −2.451, *p* = 0.014), externalizing (*Z* = −2.598, *p* = 0.009), and total problem scores (*Z* = −2.463, *p* = 0.014), on parent reports (CBCL) for 6–18-year-old children. This was also found on the subscale somatic complaints (*Z* = −2.923, *p* = 0.003). Children with only BLS had significant higher problems scores, implying worse psychological functioning. Seventy-two percent of these children had an out-of-hospital CA.

Gender was significantly related with psychopathology on the teacher reports (C-TRF); boys (1.5–5 years) showed more internalizing (*Z* = −2.018, *p* = 0.044), externalizing (*Z* = −2.580, *p* = 0.010), and total problems (*Z* = −2.745, *p* = 0.006) than girls. On self-reports (YSR), girls reported significantly more internalizing problems (*Z* = −2.196, *p* = 0.028) than boys.

Age at follow-up was a significant predictor of somatic complaints (*ρ* = 0.588, *p* = 0.044) on the TRF. For none of the questionnaires or age categories, a significant relation was found between emotional and behavioral problems and location of arrest, CA-related pre-existing condition, present health status, or SES.

## Discussion

This is the first study that addresses long-term emotional and behavioral problems in a relatively large consecutive series of children and adolescents surviving CA. Overall, the outcome after CA is reasonably well. Compared to normative data, CA survivors showed significantly more long-term attention problems and somatic complaints, on parents’ and teachers’ reports. On self-reports, significantly less social problems were found. According to parents, children showed more often psychopathological problem behavior.

Gender, age, and BLS showed significant associations with long-term outcome. Remarkably, less social problems (self-reports) but no elevated levels of anxiety, depression or posttraumatic stress problems, or associations with location of arrest or present health status were found.

### Emotions and behavior

Elevated levels on the subscale somatic complaints were found in 6–18-year-old children, according to parents and teachers. It is unknown whether these complaints were also found by Morris et al. [16]. In other groups of critically ill children, such as children after neonatal extracorporeal membrane oxygenation (ECMO) or after meningococcal septic shock, long-term somatic complaints (parent- or self-reported) are well-known [11, 27]. These results are also found in children with a chronic illness, like ConHD, and seem to be a more generic finding for chronic diseases in childhood [20]. However, we did not find elevated somatic complaints in preschool children.

For a broad age range (preschoolers, school children, and adolescents), parents and teachers reported significantly more attention problems in CA survivors. In contrast to our findings, Morris et al. described more hyperactivity-related problems [16]. Evidence for long-term attention problems after hypoxic-ischemic brain injury is mainly limited to children with ConHD and neonatal asphyxia. Long-term follow-up of children with ConHD showed overall attention problems and both inattention and hyperactivity problems 5 to 10 years after cardiac surgery [19, 20]. Studies into neonatal encephalopathy reported significantly more attention problems, or increased risk of attention deficit/hyperactivity disorder-related diagnoses at school age, compared with normative data [11, 17, 25]. Hyperactivity and inattention were found to co-exist or causally related, as described by Marlow et al. in children without physical disabilities 7 years after severe neonatal hypoxic encephalopathy [13]. However, it is difficult to generalize these findings to our patient population, as age at which hypoxia occurred is different, and comorbid underlying condition in our population is heterogeneous. As inattentional problems could be an expression of sustained attentional problems, these outcomes also suggest more neuropsychological problems.

Sustained attention is related to multiple areas in the brain, e.g., frontal lobe, prefrontal region, subcortical region, and the parietal lobe. As sustained attention is one of the basic functions of the brain, damage to the brain could lead to the failure of this function. However, neuropsychological evaluation of the present sample with the Test of Everyday Attention for Children (TEA-Ch) did not show any significant impairments on sustained attention [26]. As the TEA-Ch is a task with verbal instructions and most weaknesses were found on visual functioning, the presence of visual sustained attentional problems will be more likely. More specific neuropsychological evaluation should be done to determine the nature of these attentional problems.

According to parents, childhood CA survivors showed more often psychopathological problem behavior in comparison with the general population. In contrast, teachers and self-reports of the older CA children did not show elevated percentages of problem behavior. Due to the small number of respondents, it is difficult to draw strong conclusions. Limited awareness of one’s own emotional and behavioral functioning due to frontal lobe lesions might contribute to a potential underreporting of emotional and behavioral problems and to the positive finding on the social problem scale [26]. This has also been suggested in children after traumatic brain injury (TBI) where adolescents display less insight into executive functioning difficulties [18, 30]. On the other hand, concepts such as posttraumatic growth could also have influenced these positive outcomes on social functioning [21]. Posttraumatic growth is “the experience of positive change that occurs as a result of the struggle with highly challenging life crises” [21]. Children may worry less about futilities and may appreciate their capabilities more.

Remarkably, no elevated levels of anxiety and depression were reported, which is in contrast with long-term survivors of TBI or ConHD [10, 28]. However, long-term follow-up of other groups of critically ill children (e.g., neonatal ECMO, meningococcal septic shock) showed similar results as the present study [11, 27]. In older children, posttraumatic growth or emotional resilience could contribute to these outcomes. One could also hypothesize that a long follow-up interval could decrease complaints such as anxiety and depression. However, no association was found.

#### Predictors of outcome

Univariable analyses were limited by the small number of respondents; therefore, no strong conclusions can be drawn. However, there are some hypothesis-generating findings.

Overall, age could be an important determinant of outcome. Anderson states that “early brain damage may have a cumulative effect on ongoing development, with increasing deficits emerging through childhood as more functions are expected to mature and need to be subsumed within the undamaged tissues” [3]. This “growing into deficits” phenomenon may have an important influence on future psychological functioning [1]. As the median age at follow-up was relatively young, more problems on higher cognitive functions, such as executive functioning which includes emotional regulation, may emerge later in life, as these functions mature in adolescence [1, 26].

Additionally, in the 1.5–5-year group, older age at ICU admission gave significantly more externalizing problems and total problems. Children may work through traumatic experiences at this nonverbal age range by more externalizing behavior (screaming, shouting).

Present health status was not associated with any of the outcome measurements. Although more somatic complaints were found, they were not associated with the presence of actual comorbidities at the time of assessment, as the subscale somatic complaints contains mostly questions on physical disabilities without known medical cause.

Further, children with BLS had significantly more internalizing, externalizing, and total problems. As BLS is considered to have less impact than APLS, there might be less attention for its impact on the child’s psychological functioning. As the problems are so widespread, multidisciplinary outpatient follow-up should be organized as standard of care.

Lastly, gender was significantly related to emotional and behavioral functioning. The pattern that boys show more externalizing problems is also well-known in the general community [2]. However, in contrast to the general community, boys with CA had more internalizing problems than girls.

### Limitations

First, this is a single center cohort study with a relatively small and heterogeneous patient group. However, since this field of research is neglected, with no previous studies on this subject, this sample seems satisfactory. Second, since participants with high SES are relatively overrepresented, and some nonparticipants refused participation for emotional reasons, our results might be too positive [4, 6]. Third, correction for multiple testing was not applied since this is an explorative and descriptive study. We did not want to miss any influences on long-term outcomes. Finally, some important variables are lacking, such as time to ROSC, severity of underlying illness, treatment/course after ROSC during ICU admission, and health status at discharge. For future research, we recommend to collect these data.

### Conclusion

This is the first systematic study on long-term emotional and behavioral outcome in survivors of CA in childhood. Overall, the psychological outcomes of CA are remarkably well, as no elevated levels of anxiety, depression, or posttraumatic problems were found. However, compared with normative data, significantly more long-term somatic complaints were found. This seems to be a common finding in children after hospitalization, regardless of the illness. Further, significantly more attention problems were found.

As deficits in emotion and behavior have a significant impact on the child, their family, and society as a whole, a structured (neuro)psychological follow-up, preferably with neuroimaging, is warranted.

## Electronic supplementary material

Below is the link to the electronic supplementary material.

ESM 1 (DOC 51 kb)

## Abbreviations

APLS: Advanced pediatric life support
BLS: Basic life support
CA: Cardiac arrest
CBCL: Child Behavior Checklist
C-TRF: Caregiver-TRF
ConHD: Congenital heart disease
CPR: Cardiopulmonary resuscitation
ES: Effect size
NICU: Neonatal intensive care unit
PICU: Pediatric intensive care unit
ROSC: Return of spontaneous circulation
SES: Socioeconomic status
TBI: Traumatic brain injury
TRF: Teacher’s Report Form
YSR: Youth Self-Report
