# Supplementary material for: Cardiac arrest in infants, children, and adolescents: long-term emotional and behavioral functioning
Source: Eur J Pediatr. 2016 May 14;175:977–86. doi: 10.1007/s00431-016-2728-4 (PMC4908156; doi:10.1007/s00431-016-2728-4)
Supplement: Supplementary file 1 — (DOC 51 kb) [file 431_2016_2728_MOESM1_ESM.doc]

| Supplemental Table 1. Univariate predictors of emotional and behavioral functioning | | | | | | | | |
| --- | --- | --- | --- | --- | --- | --- | --- | --- |
| Risk factor variables | Gender | Age at ICU | BLS/APLS | Location | CA-related Pre-existing disease | SES | Age at follow-up | Present health status: Actual comorbidity |
|  | Z | ρ | Z | Z | Z | χ^2^ | ρ |  |
| *CBCL 1.5-5 yrs.* |  |  |  |  |  |  |  |  |
| Internalizing problems | - | - | - | - | - | - | - | - |
| Externalizing problems | - | .505* | - | - | - | - | - | - |
| Total problems | - | .509* | - | - | - | - | - | - |
|  |  |  |  |  |  |  |  |  |
| *CBCL 6-18 yrs.* |  |  |  |  |  |  |  |  |
| Internalizing problems | - | - | -2.451* | - | - | - | - | - |
| Externalizing problems | - | - | -2.598** | - | - | - | - | - |
| Total problems | - | - | -2.463* | - | - | - | - | - |
|  |  |  |  |  |  |  |  |  |
| *C-TRF 1.5-5 yrs.* |  |  |  |  |  |  |  |  |
| Internalizing problems | -2.018* | - | - | - | - | - | - | - |
| Externalizing problems | -2.580* | - | - | - | - | - | - | - |
| Total problems | -2.745** | - | - | - | - | - | - | - |
|  |  |  |  |  |  |  |  |  |
| *TRF 6-18 yrs.* |  |  |  |  |  |  |  |  |
| Internalizing problems | - | - | - | - | - | - | - | - |
| Externalizing problems | - | - | - | - | - | - | - | - |
| Total problems | - | - | - | - | - | - | - | - |
|  |  |  |  |  |  |  |  |  |
| *YSR 11-18 yrs.* |  |  |  |  |  |  |  |  |
| Internalizing problems | -2.196* | - | - | - | - | - | - | - |
| Externalizing problems | - | - | - | - | - | - | - | - |
| Total problems | - | - | - | - | - | - | - | - |
|  |  |  |  |  |  |  |  |  |

* p<0.05, ** p <0.01, *** p <0.001

- = not significant

Abbreviations: APLS = Advanced Pediatric Life Support; BLS = Basic Life Support; CBCL = Child Behavior Checklist; ICU = intensive care unit; SES = Socioeconomic status; (C-)TRF = (Caregiver)-Teacher’s Report Form; yrs. = years; YSR = Youth Self-Report.
